# Health insurance coverage and antenatal care services utilization in West Africa

**DOI:** 10.1186/s12913-022-07698-9

**Published:** 2022-03-07

**Authors:** Joshua Dadjo, Bright Opoku Ahinkorah, Sanni Yaya

**Affiliations:** 1 Interdisciplinary School of Health Sciences, University of Ottawa, Ottawa, Ontario Canada; 2 School of Public Health, Faculty of Health, University of Technology Sydney, Sydney, NSW Australia; 3 School of International Development and Global Studies, University of Ottawa, Ottawa, Ontario Canada; 4 The George Institute for Global Health, Imperial College London, London, UK

**Keywords:** Health insurance; primary care, Health expenditure, West Africa, Health systems, Maternal health

## Abstract

**Background:**

In recent decades, there has been a significant focus towards the improvement of maternal mortality indicators in low-and middle-income countries. Though progress has been made around the world, West Africa has maintained an elevated burden of diseases. One proposed solution to increasing access to primary care services is health insurance coverage. As limited evidence exists, we sought to understand the relationship between health insurance coverage and at least four antenatal care (ANC) visits in West Africa.

**Methods:**

Demographic and Health Survey data from 10 West African countries were weighted, cleaned, and analysed. The total sample was 79,794 women aged 15 to 49 years old were considered for the analysis. Health insurance coverage was the explanatory variable, and the outcome variable was number of ANC visits. The data were analysed using binary logistic regression. The results were presented using crude and adjusted odds ratio (aOR) at 95% confidence interval.

**Results:**

Approximately 86.73% of women who were covered by health insurance had four or more ANC visits, compared to 55.15% for women without insurance. In total, 56.91% of the total sample attended a minimum of four ANC visits. Women with health insurance coverage were more likely to make the minimum recommended number of ANC visits than their non-insured-peers (aOR [95% CI] =1.55 [1.37–1.73]).

**Conclusion:**

Health insurance is a significant determinant in accessing primary care services for pregnant women. Yet, very few in the region are covered by an insurance scheme. In the wake of the COVID-19 pandemic, policy makers should prioritize rapid solutions to provide primary care while setting the infrastructure for long-term and sustainable options such as publicly run health insurance schemes.

## Background

In September 2000, the United Nations signed the Millennium Development Goals (MDGs), which included the reduction of child mortality and improving maternal health by 2015 [1]. The MDG investments led to the decrease of the maternal mortality ratio (MMR) and under-five deaths by more than half [1]. Additionally, skilled birth assistance rose by 59% between 1990 and 2014, though only half of women received the recommended antenatal care services [1]. To build on these gains, the international community pursued the 17 Sustainable Development Goals (SDGs). Specifically, the third SDG, *Ensure Healthy Lives and Promote Well-Being at All Ages* [2] establishes the objectives of reducing under-five mortality to less than 25 in 1000, neonatal mortality to less than 12 in 1000 and MMR to 70 per 100,000 live births. So far, billions have been pledged or invested towards these goals, including a $1.4 billion commitment over 10 years by the Canadian Government [3]. Per the UN’s progress update on the SDGs in 2020, member states were not on track to meet the targets for the third goal prior to the COVID-19 pandemic [4]. It is expected that the pandemic will cause further delays in progress and threaten decades of advances [4].

Despite the decades of investment, Western Africa is known to have the world’s highest MMR [5] and one of the highest rates for under-five mortality, including at the neonatal stage [6]. The majority of deaths could be prevented with low-tech, cost-effective technologies available during facility-based delivery or in the presence of skilled birth attendants [7]. For mothers, deaths are often caused by hemorrhage, exacerbation of pre-existing conditions by pregnancy, eclampsia and sepsis [8]. For children, specifically newborns, the main cause of death is complications during birth, such as intrapartum events, preterm births, or infections. Preventative services would be key in strengthening outcomes for mothers. Yet as noted in a few studies, less than a third of women attend the minimal recommended number of antenatal services in sub-Saharan Africa [9]. Up until 2016, the World Health Organization (WHO) recommended women make at least four antenatal care (ANC) visits, though this recommendation was raised to eight visits in 2016 [10].

Universal health coverage (UHC) is the ability for people to access and use all necessary health services at a substantial quality without incurring catastrophic financial costs [11]. Specifically equity in access, strong quality of health services and protection against financial risk are the key elements of UHC according to the WHO [12], meaning UHC should lead to increased access to essential medical services and decreased rates of catastrophic costs to consumers.

Globally, low-and middle-income (LMICs) countries have employed one of two systems: community based health insurance (CBHI), or schemes, and social or national health insurance [13]. CBHIs tend to focus on covering those who are not covered by other schemes [13] by taking a resource pooling approach that uses social structures such as families, community groups or religious groups [14]. CBHI is understood to cause a greater use of outpatient services without increases in inpatient services, lower rates of community health expenditure including lower out-of-pocket costs, higher use of services for children under five and an overall improvement in health indicators such as immunization rates and under-five mortality [14]. Social health insurance schemes are commonly found in the developed world, and have recently been implemented in LMICs such as Ghana [15] and Nigeria [16]. Unfortunately, these systems are often weakened by a lack of institutional capacity and small tax bases, therefore limiting the possibility of attaining full coverage [17]. For example, Nigeria’s system has been hurt by poor financial management, including insufficient funding and a weak financial safety net for the poor [18]. Ghana’s system has a premium cost setting system that has excluded many low-income families [19]. These are two examples of the challenges that many LMICs face. Other challenges are the inadequate distribution of services, the inability of systems to evolve to deal with complications related to pregnancy, insufficient staffing and equipment [20]. As some studies have shown, insurance membership is associated with a greater likelihood of accessing services, yet barriers, as, for example, the distance to services, have been found to limit the use of services and therefore, maintain high mortality rates for women who go unassisted [19].

This study adopts the Health Care Services Utilization Model put forward by Andersen to consider the relationship between health insurance coverage and number of ANC visits [21]. This model was similarly adapted for a study related to maternal health care in Jordan [22]. This model identifies three types of factors: predisposing factors which are factors such as demographics and social structures; enabling factors such as income or insurance status; and need for care factors which consider how one views their health and understands their need for care [22].

There is much evidence on the association between increased health insurance coverage and access to maternal health services globally, yet few studies have specifically considered West Africa. This region is worthy of unique consideration due to its elevated burden of disease and the lack of progress that has been made in improving child and maternal health indicators [6, 8]. In turn, UHC is believed to help lower barriers to access for basic, life-saving primary care services such as antenatal care [19]. It would be valuable for policy makers within the international development community to consider if the impact of increased health coverage in this unique region is sufficient because the elevated and persistent burden of disease may indicate that UHC without additional efforts in other areas is insufficient. Therefore, this study seeks to fill the gap in the literature by using data from the most recent Demographic and Health Surveys of 10 West African countries, to observe the relationship between health insurance coverage and number of antenatal care visits for mothers.

## Methods

### Study design

We pooled data from the women’s files of the most recent (2010–2019) Demographic and Health Surveys (DHS) of 10 West African countries (Benin, Côte d’Ivoire, Ghana, Gambia, Guinea, Liberia, Mali, Niger, Nigeria, Togo) that are part of the DHS programme and for whom data has been published in the past decade. The DHS are nationwide surveys of LMIC collected every 5 years. They are cross-sectional and gather information on health and other population characteristics. The DHS adopts a two-stage stratified sampling technique to collect nationally representative data from the respondents. The two-stage sampling process begins with the selection of clusters usually called enumeration areas (EAs). This is followed by the selection of households for the survey. For this study, data came from DHS’ questionnaires for women. This is a standard model questionnaire that has been applied throughout the globe [23]. The sample size was 79,794 participants. Data in the form of datasets can be accessed through the DHS website. The selected countries had the relevant maternal health data needed for this study. The methods used by the DHS in carrying out the surveys were followed in conducting this study.

### Variable

#### Outcome variable

The outcome variable was number of ANC visits. The exact question in the DHS questionnaire was “how many times did you receive antenatal care during this pregnancy?” [24, 25]. Respondents either gave a number or indicated they did not know. Less than four ANC visits was coded as “0” and four or more was coded as “1”. At least four ANC visits was the outcome of interest in this study.

#### Explanatory variable

The main explanatory variable was health insurance coverage. The question asked was “are you covered by any health insurance?” Respondents answered yes or no [24, 25] and since this data is binary, no further coding was needed.

### Covariates

Ten variables linked to various socio-economic and demographic indicators were added as covariates based on the findings of previous studies [26–29]. These were age, marital status, education, place of residence, wealth index, birth order, sex of head of household, frequency of reading newspaper, frequency of listening to the radio, and frequency of watching television. Age ranges were 15–24, 25–29, 30–34, 35–39, 40–44 and 45–49 years old. Marital status was coded as not married, married, cohabitating, widowed, divorced, and no longer living together. Educational levels were no education, primary, secondary, and higher education. The wealth index was a quintile of five categories: poorest, poorer, middle, richer, and richest. The birth order was re-coded into two categories: 1 to 3 and 4 or more. The sex of head of household was either male or female and the place of residence was urban or rural. Exposure to radio, television or newspaper were divided in four categories: not at all, less than once a week, at least once a week and almost every day.

### Statistical analysis

We used Stata version 16.1 for Windows. Data for the 10 countries were pooled to ease the analysis and interpretation. A weighting factor ($\frac{v005}{1000000}$) was applied to adjust for over and under sampling. The pooled and weighted data with a sample size of 81,099 was cleaned to drop missing cases from the outcome variable (*n* = 1158, % = 1.43%) and explanatory variables (*n* = 147, % = 0.18). This was done because the analysis was based on complete observations for all the variables of interest. The analysis was descriptive and multivariable. For the descriptive analysis, a *X*^2^ test was used to express the relationship between the outcome and explanatory variables. The multivariable analysis was conducted on all the explanatory variables. We conducted a bivariate (Model I) and multivariable (Model II) logistic regression, alongside their adjusted odds ratio with their corresponding confidence interval (95%).

## Results

### Health insurance coverage and minimum of four ANC visits

**Fig. 1:**
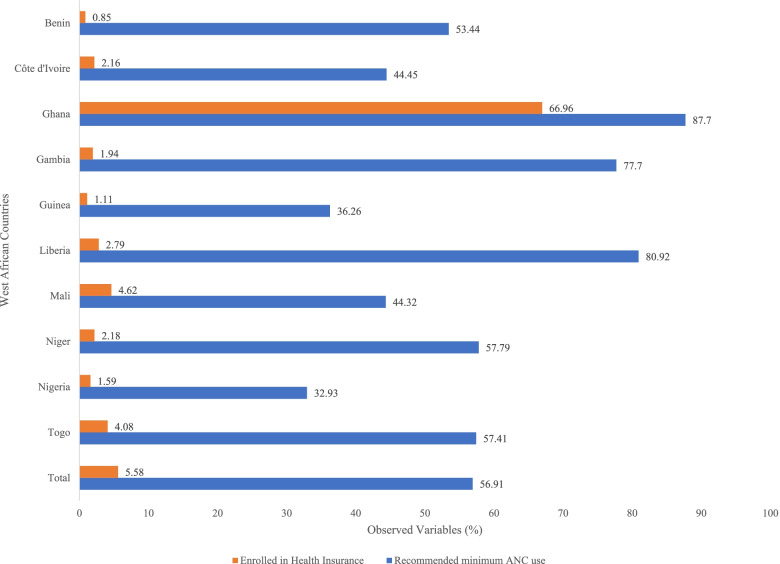
Recommended ANC visits and Health Insurance Coverage in 10 West African Countries

Table 1 presents the results showing participants with a minimum of four ANC visits according to the explanatory variables. The total sample was 79,794 women aged 15–49 years. Approximately 86.73% of women who had health insurance had the number of recommended minimum ANC visits, compared to 55.15% for women without insurance. In total, 56.91% of the total sample attended a minimum of four ANC visits. The highest prevalence of four or more ANC visits was in Ghana (87.7%) and the lowest prevalence in Nigeria (32.93%). The overall prevalence of health insurance coverage was 5.58%, with the highest in Ghana (66.96%) and the lowest in Guinea (1.11%) (see Fig. 1).”

**Table 1:** Minimum of four ANC visits by explanatory variables (*n*= 79,794)

Variables	Weighted N	Weighted %	***P***-value	X^2^
**Health Insurance Coverage**			<0.001	1700
No	41,554	55.15		
Yes	3,864	86.73		
**Age (in years)**			<0.001	189.21
15-19	2805	50.85		
20-24	9048	55.25		
25-29	11913	57.43		
30-34	9923	59.42		
35-39	7197	58.92		
40-44	3382	56.37		
45-49	1147	51.11		
**Marital Status**			<0.001	387.86
Not married	2376	63.34		
Married	35710	55.04		
Cohabiting	5255	65.53		
Widowed	534	61.3		
Divorced	597	63.04		
No longer living together	938	70.88		
**Educational Level**			<0.001	6900
No education	19985	44.24		
Primary	8812	63.55		
Secondary	13784	78.15		
Higher	2831	91.02		
**Wealth Index**			<0.001	4500
Poorest	6611	39.38		
Poorer	8066	47.86		
Middle	9120	56.17		
Richer	10401	66.04		
Richest	11214	79.18		
**Birth Order**			<0.001	563.24
1 to 3	26655	61.57		
4 or more	19759	51.83		
**Sex of Head of Household**			<0.001	462.34
Male	37151	55.25		
Female	8252	65.82		
**Place of Residence**			<0.001	3800
Urban	21463	72.53		
Rural	23950	47.71		
**Exposure to Newspaper**			<0.001	1700
Not at all	39347	54.35		
Less than once a week	3734	81.64		
At least once a week	2302	82.96		
Almost every day	29	71.02		
**Exposure to Radio**			<0.001	3200
Not at all	13296	44.28		
Less than once a week	12148	61.05		
At least once a week	19132	66.9		
Almost every day	837	66.06		
**Exposure to Television**			<0.001	4600
Not at all	19742	45.59		
Less than once a week	8855	63.85		
At least once a week	16014	74.21		
Almost every day	801	77.21

### Socio-demographic characteristics and making the minimum of four ANC visits

Accessing the minimum recommended ANC services increased slightly per age bracket, peaking at the 30–34-year age range. Only 55% of married women made the minimum number of ANC visits compared to 63.34% of non-married women and increasing from there for other non-married marital statuses. Educated women and wealthier women made the minimum ANC visits at a higher rate than their less educated and poorer counterparts. Women in urban settings made the minimum recommended visits at a significantly higher rate than rural women. Mothers made the minimum ANC visits more often for their first three children than for the fourth and additional children. There were also observable differences due to the sex of the head of household with 65.82% of women attending minimum ANC visits in homes where women are the head, versus 55.82% where men are the head. Finally, women with elevated exposure to radio, television and newspaper made their minimum recommended ANC visits at a higher rate than those with lower exposure to those mediums.

### Logistic regression analysis results on health insurance coverage and minimum ANC visits

Table 2 presents the regression analysis results. The logistic regression analysis shows that the odds of attending four or more ANC visits were higher for those with insurance than those without (aOR [95% CI] =1.55 [1.37–1.73]). We also found that 30- to 34-year-old women were more likely to make the minimum ANC visits then their younger or older peers (aOR [95% CI] =1.43 [1.32–1.54]). Married women had the lowest odds of attending the minimum recommended ANC visits (aOR [95% CI] =1.44 [1.32–1.57]), compared to women who were cohabitating (aOR [95% CI]) =1.45 [1.31–1.59]) or no longer living with their spouse (aOR [95% CI] =1.40 [1.19–1.61]). Regarding education, more educated women had higher odds of making the recommended visits with women with post-secondary education having the strongest odds (aOR [95% CI] = 4.03 [3.45–4.60]). The richest women were more like to make the required minimum ANC visits (AOR [95% CI] =3.31 [3.07–3.56]) than poorer women (aOR [95% CI] =1.38 [1.31–1.44]). For the fourth or more births, the odds off making the minimum number of visits was lower (aOR [95% CI] =0.82 [0.78–0.85]) than for the first to third birth. Homes where heads of households are women were more likely to make the minimum ANC visits (aOR [95% CI] =1.14 [1.08–1.19]) than homes with men as the head of household. Women in rural settings were less likely to make the recommended number of ANC visits (aOR [95% CI] =0.97 [0.93–1.02]) then their urban counterparts. Furthermore, any exposure to newspapers, radio or television increased odds of making the minimum number of visits, with small variations between the levels of exposure. Finally, making the minimum recommended ANC visits was highest in Ghana (aOR [95% CI] =3.96 [3.46–4.46]) and lowest in Côte d'Ivoire (aOR [95% CI] =0.37 [0.31–0.43]).

**Table 2:** Logistic regression results on the association between health insurance coverage and four or more ANC visits

Variables	Model I cOR	95% CI		***p***-value	Model II aOR	95% CI		***p***-value
**Health Insurance Coverage**								
No **(reference)**								
Yes	5.03	4.61	5.45	<0.001	1.55	1.37	1.73	<0.001
**Age (in years)**								
15-19 **(reference)**								
20-24	1.20	1.13	1.28	<0.001	1.06	0.99	1.13	0.12
25-29	1.31	1.23	1.38	<0.001	1.23	1.14	1.32	<0.001
30-34	1.41	1.32	1.50	<0.001	1.43	1.32	1.54	<0.001
35-39	1.42	1.33	1.51	<0.001	1.54	1.41	1.66	<0.001
40-44	1.23	1.14	1.32	<0.001	1.49	1.35	1.62	<0.001
45-49	1.08	0.98	1.19	0.12	1.50	1.33	1.67	<0.001
**Marital Status**								
Not married **(reference)**								
Married	0.74	0.69	0.79	<0.001	1.44	1.32	1.57	<0.001
Cohabiting	1.08	0.99	1.17	0.07	1.45	1.31	1.59	<0.001
Widowed	0.91	0.78	1.04	0.16	1.47	1.22	1.71	<0.001
Divorced	0.89	0.76	1.02	0.09	1.22	1.01	1.43	0.03
No longer living together	1.32	1.15	1.49	<0.001	1.40	1.19	1.61	<0.001
**Educational Level**								
No education **(reference)**								
Primary	2.13	2.05	2.22	<0.001	1.55	1.48	1.62	<0.001
Secondary	4.04	3.88	4.20	<0.001	2.09	1.98	2.20	<0.001
Higher	11.75	10.26	13.24	<0.001	4.03	3.45	4.60	<0.001
**Wealth Index**								
Poorest **(reference)**								
Poorer	1.41	1.35	1.46	<0.001	1.38	1.31	1.44	<0.001
Middle	1.89	1.81	1.97	<0.001	1.75	1.67	1.84	<0.001
Richer	2.61	2.50	2.73	<0.001	2.29	2.16	2.42	<0.001
Richest	4.55	4.33	4.78	<0.001	3.31	3.07	3.56	<0.001
**Birth Order**								
1 to 3 **(reference)**								
4 or more	0.71	0.69	0.73	<0.001	0.82	0.78	0.85	<0.001
**Sex of Head of Household**								
Male **(reference)**								
Female	1.55	1.49	1.61	<0.001	1.14	1.08	1.19	<0.001
**Place of Residence**								
Urban **(reference)**								
Rural	0.41	0.40	0.43	<0.001	0.97	0.93	1.02	0.21
**Exposure to Newspaper**								
Not at all **(reference)**								
Less than once a week	3.44	3.17	3.70	<0.001	1.08	0.99	1.18	0.10
At least once a week	3.68	3.31	4.05	<0.001	0.92	0.81	1.03	0.13
Almost every day	2.19	0.88	3.50	0.11	0.64	0.25	1.03	0.06
**Exposure to Radio**								
Not at all **(reference)**								
Less than once a week	1.99	1.92	2.06	<0.001	1.32	1.27	1.38	<0.001
At least once a week	2.48	2.39	2.56	<0.001	1.39	1.33	1.45	<0.001
Almost every day	2.33	2.05	2.60	<0.001	1.39	1.33	1.45	<0.001
**Exposure to Television**								
Not at all **(reference)**								
Less than once a week	2.03	1.95	2.11	<0.001	1.13	1.07	1.18	<0.001
At least once a week	3.14	3.02	3.25	<0.001	1.26	1.20	1.33	<0.001
Almost every day	3.94	3.33	4.55	<0.001	1.26	1.03	1.49	0.02
**Country**								
Benin **(reference)**								
Côte d'Ivoire					0.37	0.31	0.43	<0.001
Ghana					3.96	3.46	4.46	<0.001
Gambia					3.26	3.03	3.48	<0.001
Guinea					0.51	0.47	0.55	<0.001
Liberia					3.47	3.18	3.76	<0.001
Mali					0.61	0.57	0.65	<0.001
Nigeria					1.09	1.03	1.15	0.002
Niger					0.42	0.39	0.45	<0.001
Togo					1.07	0.98	1.16	0.156

## Discussions

In this study, we examined the relationship between health insurance coverage and making the recommended number ANC visits in 10 West African countries for whom recent DHS data was available. We also looked at other socio-demographic characteristic to study their relationship with making ANC visits. Our results show that women with health insurance have greater odds of making their recommended number of visits than their non-insured counterparts. This is likely because insurance provides sufficient protection from catastrophic expenditure and because insurance can be linked to other socio-economic indicators, such as wealth and education, that are known determinants of ANC use [26]. This finding is comparable to that of previous studies of the association of insurance on ANC use in LMICS [13, 19, 30, 31], where researchers found that insurance can meaningfully lower catastrophic cost to make services more accessible, though barriers, such as premiums, can limit its overall impact. As only 5.6% of women have health insurance, protection against catastrophic expenditure through insurance is seemingly limited to a select few. Of note, most women without health insurance are still able to make the minimum required ANC services. These findings suggest there are other, more affordable strategies to make ANC services more accessible.

Past studies provide further confirmation for our findings. Wang, Temsah and Mallik published a DHS analytical study in 2014 that considered the impact of health insurance on maternal health care utilization in LMICS [13]. They reported that health insurance had an overall positive impact on access to various maternal health services. Specifically, they reported that insurance positively affected initiating antenatal care in the first trimester in Namibia, Indonesia, and Burundi. It would stand to reason that as insurance would promote the initiation of ANC, it would contribute to making the recommended number of visits. In another contribution, the same authors speak to evidence from Ghana, Rwanda and Indonesia [19]. There again, their results showed a positive association between health insurance and utilization of maternal health services. In Ghana and Indonesia, insurance was linked to making the recommended number of ANC visits, and in Rwanda, it was associated with making at least one visit. In addition, Comfort, Peterson and Hatt [30] conducted a systematic review of the evidence on health insurance and its effects on the use and provision of maternal health services and on maternal and neonatal health outcomes in LMICs and found that the many studies that focused on health insurance and service use provided consistent evidence of a positive correlation between insurance and use of services. Finally, Abdulai and Adams examined a local example in northwestern Ghana. Again, they concluded that insurance improved access and utilization of maternal health services [31]. These studies confirm and endorse our findings while acknowledging the impacts of the makeup of various schemes and different socio-economic barriers facing those seeking care.

This study has important implications for international health policy. As noted in the 2020 progress report [4], though global gains were being made, the rate of progress was not enough to satisfy the targets of SDG goal 3. This progress was further stunted by the COVID-19 pandemic and its straining effects on health system throughout the world. As the purpose of these targets is to avoid deaths that would not occur if basic primary health services were available, it is imperative the international community reorient itself to achieve the targets of SDG 3, by prioritizing policies and strategies that would have rapid and immediate impacts.

This study provides some insight on how to re-orient those efforts. First, most women do not have health insurance, yet within that population, a majority still access recommended ANC services. This suggest other strategies to reduce cost, such as capping or eliminating user-fees, may be more accessible and effective to a broader number of women. Studies have found that reducing or eliminating user fees help the poorest and least educated the most [32], those who according to this study, are the least likely to make the recommended number of ANC visits. This may occur because these strategies require less infrastructure and less effort on behalf of patients who do not need to take administrative steps such as registering. These strategies are also more likely to be adopted and operated at local levels and easily stood up in areas of acute needs. In fact, evidence has shown that the introduction or removal of user fees have immediate and abrupt impact on health services utilisation [33]. Our findings indicate that women who were exposed to radio, television or newspapers on a regular basis were more likely to make the recommended number of ANC visits. Therefore, these channels of public communication could be used to raise awareness concerning changes in policy.

Furthermore, as noted earlier, evidence in this study on the association between marital status or household gender with ANC visits, suggest that efforts to lower catastrophic expenditure should be paired with efforts to educate men alongside women on the need to access primary care services during pregnancy. The experience of organizations such as Médecins Sans Frontières [34] in managing these dynamic health systems would be invaluable in seeking rapid gains in the post-COVID world.

These proposed solutions could have immediate impact, but there is little evidence of their long-term sustainability [33]. A public health insurance scheme is a long-term solution that has proven its sustainability throughout the world, and has had positive results in Ghana [35]. West African countries will face several barriers to setting up a public health insurance system that is equitable. The experience of Ghana and Nigeria suggest that poor institutional capacity due to limited financial resources and mismanagement will be among those barriers [18, 19]. Building capacity in these areas will be crucial in building the health systems that will provide meaningful access to services for the most vulnerable women in the region. Therefore, major global health institutions should focus on helping countries build their capacity to manage their public system. Finally, closing the wealth inequality gap that exist in West Africa [36] will be essential to building the capacity of health systems. One approach is to reform tax policies and strengthen collection systems. In doing so, countries would secure more financial resources and allow greater redistribution into social programs benefiting the poor, including health insurance.

### Strengths and limitations

This multi-country analysis used comparable data to examine health insurance coverage and its association with number of ANC visits. Our findings confirm several other studies while providing novel information. Our main limitation is there is limited recent data for this region of the world. Therefore, our findings depend on only 10 countries in West Africa, including the richer countries and may ignore some realities from poorer countries. Our findings would be strengthened if data from more country became available. Another limitation is the cross-sectional nature of the study design used that made in impossible to establish causality.

## Conclusion

In conclusion, this study indicates that in West Africa, women with health insurance have greater odds of making the minimum ANC visits, though only 5.6% of women in the sample have insurance. This study also suggest that health insurance is an important determinant of accessing ANC services, though other factors are to be considered. Future studies should seek to establish the determinants of health insurance coverage as to assess if coverage is equitably accessed throughout West Africa. Specifically, because this study found such strong links between ANC access and education, future studies should examine education levels among insured women. Future investigations could also consider whether women with health insurance are also more likely to access other primary care services such as facility-based delivery and skilled birth attendants.

## Data Availability

Data for this study were sourced from Demographic and Health surveys (DHS) and available here: http://dhsprogram.com/data/available-datasets.cfm.
